# A direct CO_2_ control system for ocean acidification experiments: testing effects on the coralline red algae *Phymatolithon lusitanicum*

**DOI:** 10.7717/peerj.2503

**Published:** 2016-09-27

**Authors:** Laura Sordo, Rui Santos, Joao Reis, Alona Shulika, Joao Silva

**Affiliations:** CCMAR–Centre of Marine Sciences, Universidade do Algarve, FARO, Portugal

**Keywords:** Control system, Ocean acidification (OA), CO_2_ bubbling, Coralline algae

## Abstract

Most ocean acidification (OA) experimental systems rely on pH as an indirect way to control CO_2_. However, accurate pH measurements are difficult to obtain and shifts in temperature and/or salinity alter the relationship between pH and *p*CO_2_. Here we describe a system in which the target *p*CO_2_ is controlled via direct analysis of *p*CO_2_ in seawater. This direct type of control accommodates potential temperature and salinity shifts, as the target variable is directly measured instead of being estimated. Water in a header tank is permanently re-circulated through an air-water equilibrator. The equilibrated air is then routed to an infrared gas analyzer (IRGA) that measures *p*CO_2_ and conveys this value to a Proportional-Integral-Derivative (PID) controller. The controller commands a solenoid valve that opens and closes the CO_2_ flush that is bubbled into the header tank. This low-cost control system allows the maintenance of stabilized levels of *p*CO_2_ for extended periods of time ensuring accurate experimental conditions. This system was used to study the long term effect of OA on the coralline red algae *Phymatolithon lusitanicum*. We found that after 11 months of high CO_2_ exposure, photosynthesis increased with CO_2_ as opposed to respiration, which was positively affected by temperature. Results showed that this system is adequate to run long-term OA experiments and can be easily adapted to test other relevant variables simultaneously with CO_2_, such as temperature, irradiance and nutrients.

## Introduction

Mesocosm systems are widely used in ocean acidification (OA) research, allowing the simulation of predicted ocean conditions for the near future. While important information has been obtained regarding the response of different kinds of marine organisms to increased CO_2_ and lower pH levels, inconsistencies and uncertainties in results are common, even among similar experiments with related taxa (Gattuso, Mach & Morgan, 2013). Some of these discrepancies may be related to the control mechanisms used to maintain the desired experimental levels (typically pH levels). A greater understanding of the complex responses of marine organisms and ecosystems to high CO_2_ requires accurate carbonate system manipulations and well-controlled experimental setups (Schulz et al., 2009).

Four parameters are often used to describe the seawater carbonate system in OA experiments: pH, CO_2_ partial pressure (*p*CO_2_), dissolved inorganic carbon (DIC) and total alkalinity (TA). From any two of these parameters it is possible to calculate the other two as well as a few other parameters of the system. However, such calculations rely on empirically derived apparent dissociation constants, which are functions of temperature and salinity (Dore et al., 2009). Because there is no consensus on which two parameters should preferentially be measured, carbonate system calculations in different OA studies are often based on different input parameters. The direct measurement of *p*CO_2_ in seawater is considered to be difficult in small volumes, and so this variable is usually calculated from pH, TA or DIC data. Therefore, real uncertainties in both measured and calculated values persist and are often unknown, especially under high *p*CO_2_ (Hoppe et al., 2012). For example, in the few datasets of OA studies where all parameters were measured, Hoppe et al. (2012) found that *p*CO_2_ values calculated from TA and DIC were about 30% lower than those calculated from TA and pH or from DIC and pH. Because of this, the calculated parameters of the carbonate system (e.g. *p*CO_2_, calcite, saturation state) are not always comparable between CO_2_ perturbation studies (see Hoppe et al., 2012).

The most used control variable in OA experiments is pH. After a long debate on the use of different calibration scales and protocols (Dickson, 1993; Rérolle et al., 2012) the total scale has been established as the most appropriate and recommended scale for pH determinations in seawater (Rérolle et al., 2012). The determination of seawater pH is intrinsically difficult, it is temperature and salinity dependant and only for solutions whose pH values closely match that of a standard can the pH be regarded as an approximate measure of the hydrogen ion activity of the solution (Gieskes, 1969). Most chemical sensors have inherent drifts and unpredictable behaviors, requiring frequent recalibrations to improve accuracy. In addition, accuracy is also affected by the type of electrode, calibration type, electrode filling solution, measurement conditions and susceptibility to electromagnetic interferences (Seiter & DeGrandpre, 2001; Dickson, 1993).

The spectrophotometric pH measurement is more accurate and precise than the potentiometric method and is being established as the reference method (see Dickson, 1993; Hoppe et al., 2010; Rérolle et al., 2012). There are already semi-automated systems that can be used to implement the standard spectrophotometric approach (Carter et al., 2013; McGraw et al., 2010). However, their accuracy is still dependent on the inherent uncertainties of spectrophotometric pH measurements such as dye impurities (see Carter et al., 2013; McGraw et al., 2010). The accuracy of the pH measurements could be improved by purifying the dye and determining the molar absorptivity ratios for the dye and spectrometer (McGraw et al., 2010), or adequately addressing the dye impurities and the indicator’s temperature and salinity (Carter et al., 2013). Even if the spectrophotometric measurement is the ideal technique to measure seawater pH, there are still difficulties to apply it to real-time pH determinations or monitoring/control systems, partially because this technique cannot be applied for high frequency (e.g. one min interval) underway measurements (Frankignoulle & Borges, 2001).

In addition, the calculation of *p*CO_2_ from pH is influenced by other variables such as salinity and, even more critically, temperature. Particularly in systems with temperature fluctuations, the necessary pH to reach a certain target *p*CO_2_ will be different for every temperature, which is naturally incompatible with the conventional pH-stats control systems. Hence, even considering that the pH readings can be accurate, these systems will always have variations of *p*CO_2_ as a function of temperature. Because the ocean’s pH is expected to decrease as a consequence of the increase in atmospheric CO_2_, it is logical that OA experiments should target the future projections of *p*CO_2_ concentrations in seawater.

The partial pressure of CO_2_ (*p*CO_2_) is a parameter commonly used in oceanography, mostly for underway measurements on board research vessels. In these systems, water is continuously pumped through a water-gas equilibrator (Frankignoulle, Borges & Biondo, 2001), where the partial pressure of CO_2_ in the water phase is equilibrated with the air and CO_2_ is then read in the gas phase by an infrared gas analyser (IRGA). These systems provide very accurate and robust measurements of seawater *p*CO_2_, which makes them particularly adequate for long-term or continuous data-acquisition setups.

In the context of high-CO_2_ research, either on terrestrial, freshwater or marine (OA) environments, *p*CO_2_ is the obvious variable used to describe present conditions and future scenarios. Abril et al. (2015) recently pointed out that regional and global estimates of CO_2_ outgassing from freshwater based on pH and TA are most likely overestimated and direct *p*CO_2_ measurements are recommended in inland waters. Therefore, it appears only logical that CO_2_ could be used not only as a simple descriptor but also as the control variable in experimental systems. If such is already happening in terrestrial high-CO_2_ research (e.g. Lin et al., 1999) and a recommendation for its use in freshwater has recently been published (see Abril et al., 2015), such is not the case yet for OA experimental systems, where pH-stats remain the most common control method.

Here we describe a technical approach to set and control *p*CO_2_ levels in an open-circuit mesocosm system using CO_2_ as the control variable. Seawater in header tanks is equilibrated with air, and CO_2_ is analyzed with a non-dispersive IRGA. This information is then transmitted to a Proportional-Integral-Derivative (PID) digital controller that in turn regulates pure CO_2_ injection in the header tanks through an algorithm-regulated process, thus avoiding fluctuations of the target *p*CO_2_ that are characteristic of all on-off processes. This semi-automated system can maintain continuous and stable *p*CO_2_ set points and does not require frequent calibrations. It is more autonomous than previous CO_2_ control systems and is suitable for running long-term experiments. The system was developed with the objective of assessing the long-term effects of OA on the coralline algae *Phymatolithon lusitanicum,* recently described as a new species for science and the most common in the mäerl beds from Southern Portugal (see Peña et al., 2015). Here we evaluated the long-term effects (11 months) of high CO_2_ in the photosynthetic rates of the algae. As well, the effects of temperature on the respiration rates of *P. lusitanicum*, at different CO_2_ levels, was assessed.

Several authors have highlighted the need for more long-term experiments to evaluate the response of coralline algae to OA (e.g. Martin et al., 2013; Ragazzola et al., 2013; McCoy & Kamenos, 2015). Long-term studies can reveal very different results with respect to short-term experiments (McCoy & Kamenos, 2015) and give important information on the potential for physiological acclimation (see Hurd et al., 2009; Martin et al., 2013; Ragazzola et al., 2013). Long-term experiments, where the synergistic effect of light, temperature and other factors is also investigated, are essential to understand how ecosystems will respond to global change.

## Methods

### Mesocosm layout

**Figure 1:**
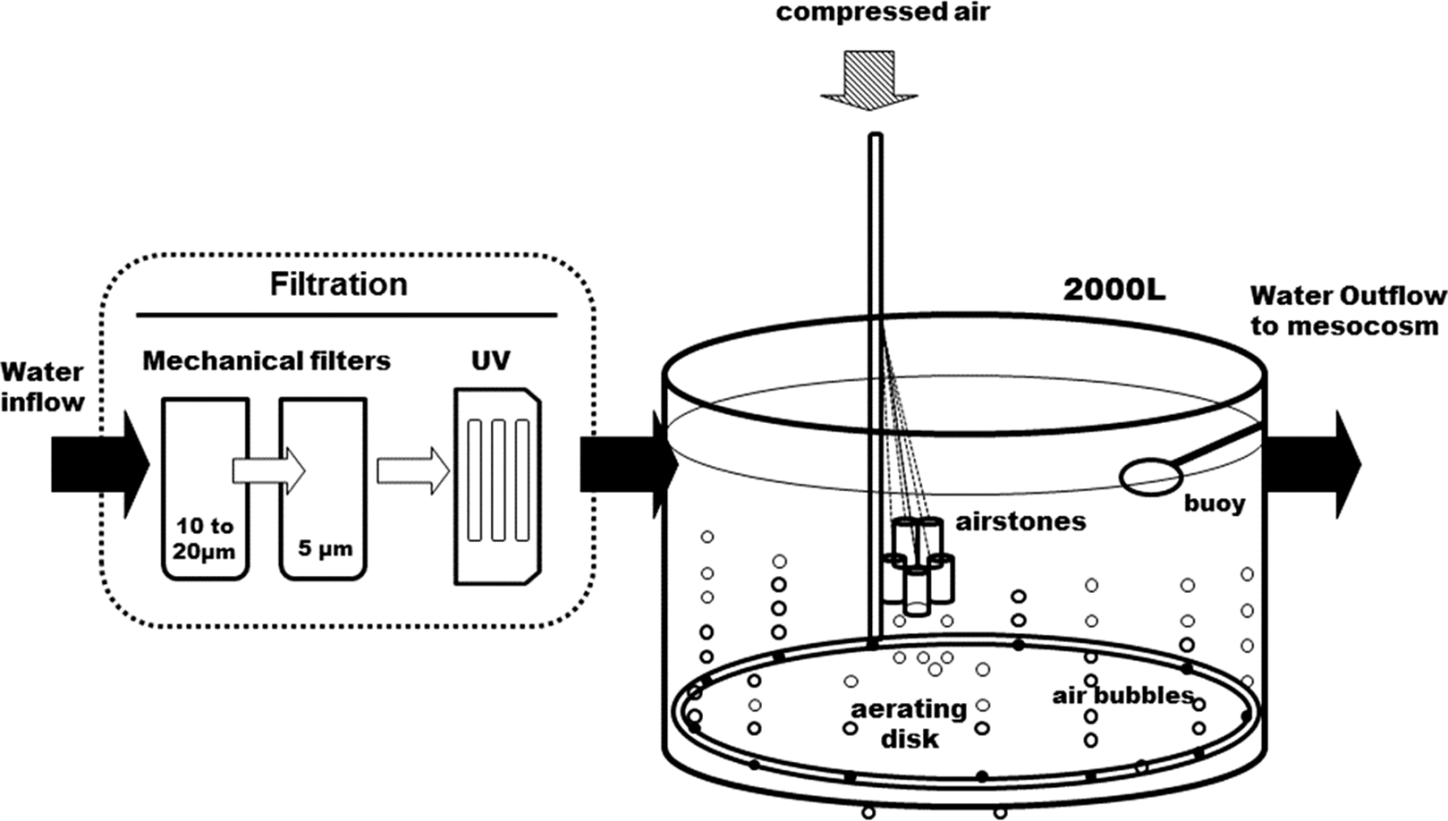
Diagram of the water supply system. The seawater is filtered before it enters the 2,000 L open tank. A large aerating ring and air stones maintain a continuous air bubbling to equilibrate seawater with air before it gets distributed into the head tanks (see Fig. 2).

The whole circuit was assembled indoors, in a temperature-controlled room. Following decantation, sand- and cartridge-filtering (10–20 and 5 μm), running seawater enters a preliminary 2,000 L tank, where it is strongly bubbled with compressed air, to insure full *p*CO_2_ equilibration with the atmosphere (Fig. 1). It is then UV-filtered (16 and 8 W) and pumped to the three 200 L header tanks of the circuit, one per *p*CO_2_ level, where CO_2_ is injected. From each header tank, water is pumped to six 25 L individual experimental aquaria, in a continuous 0.05 L min^−1^ flow. This is a flow-through open circuit. The temperature is largely maintained by the room’s air-conditioning system, with the assistance of dedicated water-chillers, when deemed necessary. While *p*CO_2_ is continuously measured in the header tanks, temperature, pH, dissolved oxygen and salinity are monitored regularly in the experimental aquaria.

### *p*CO_2_ control system

**Figure 2:**
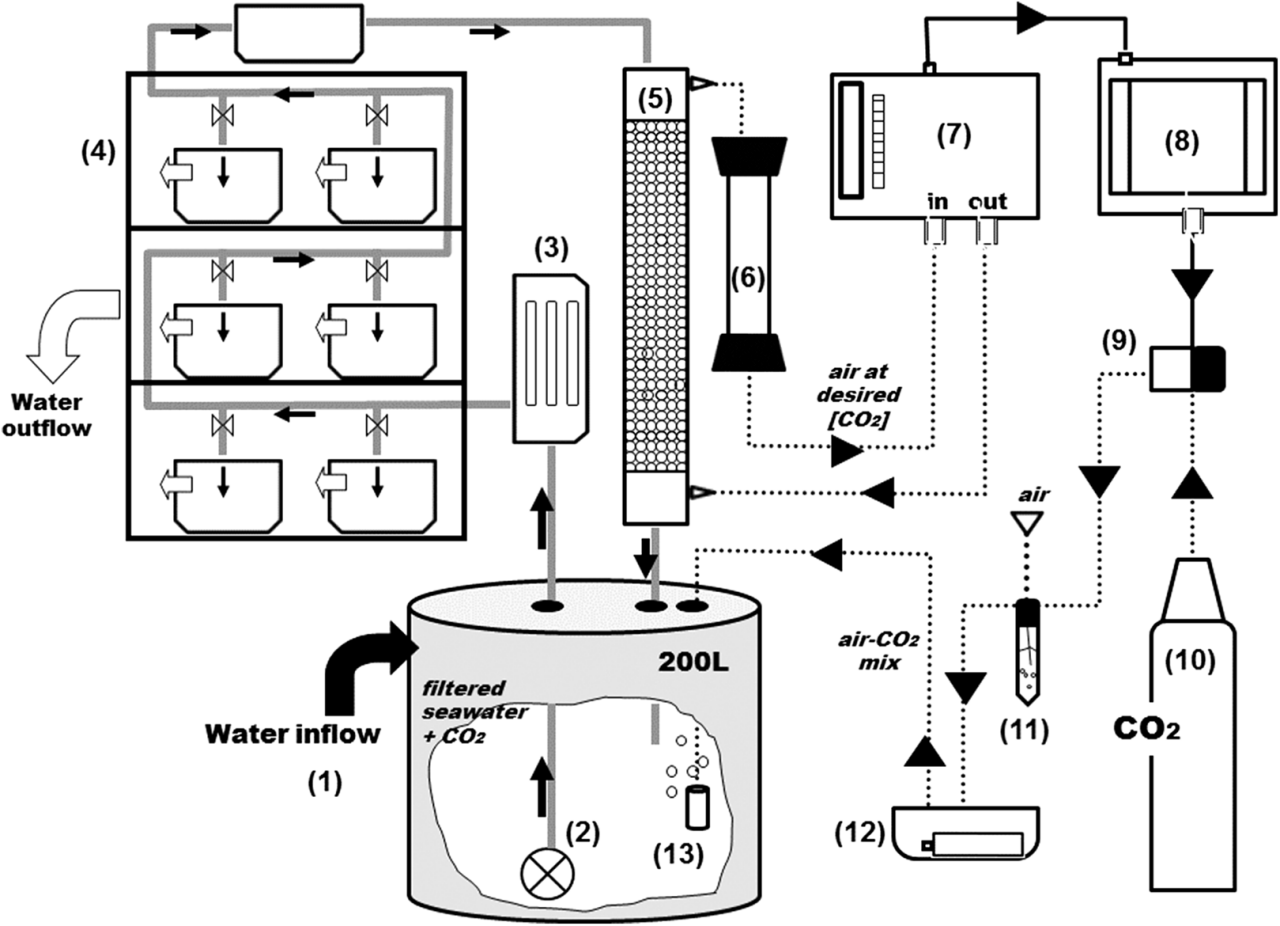
General scheme of the experimental system. Grey and black solid arrows indicate water flow and electrical connections respectively and dotted arrows indicate CO_2_-air flow. From the 2,000 L tank (see Fig. 1), water goes into a second reservoir of 200-L (1) and with a water pump (2) is pushed through an UV filter of 8 W (3) and distributed to the aquariums and a sealed box located on the top of the shelves (4). From the sealed box, seawater reaches the top of the equilibrator with a close air circuit (5) and goes through a Drierite syringe (6) before entering the analyzer. The rate of CO_2_ injection into the system is controlled by a CO_2_ gas analyzer (7) coupled to a PID controller (8) and a solenoid valve (9) which is connected to the CO_2_ bottle (10). Air and *p*CO_2_ are mixed in a falcon flask (11) and injected into the head tank using an air pump on a sealed box (12) with aquarium air stones (13).

The CO_2_ control system is depicted in Fig. 2. At the 200 L header tank (1) water is flushed with industry-grade commercial CO_2_. From the header tanks water is pumped (2) through an 8 W UV filter (3) and is distributed to the experimental aquaria (4) Part of the water is directly channeled to a gas flushing equilibrator (5). The air-flushing equilibrator is used to equilibrate gas partial pressures between water and air, and was built according to Frankignoulle, Borges & Biondo (2001). It consists of a vertical Plexiglas tube (height: 80 cm, diameter: 10 cm), sealed at both ends and filled with glass marbles to increase the exchange surface and reduce air volume. Seawater enters the equilibrator from the top (at 3 L min^−1^) and percolates downwards, being returned to the header tank. The air coming from the IRGA (7) is injected at the bottom and aspired at the top of the equilibrator, returning to the IRGA for analysis, after passing through a Drierite® column (6) for humidity scrubbing. The air that circulates between the IRGA and the equilibrator is therefore a closed circuit of low volume (∼785 ml). The CO_2_ dissolved in the water equilibrates with the air (wet air) and is directly read by the IRGA (WMA-4, PP Systems, MA, USA). All air-carrying tubing is made out Tygon® to minimize CO_2_ losses.

The PID controller (8) (TEMPATRON PID330; Tempatron, Farnell, UK) is an advanced controller, originally designed for the control of processes in a wide range of industrial applications. In this system, the PID controller receives the CO_2_ readings from the IRGA and controls the operation of an in-line solenoid valve (9) that opens and closes CO_2_ injection (10). The controller determines the difference between the measured CO_2_ and the programmed set point and attempts to minimize it by adjusting the process control input (CO_2_ injection). The PID controller also comprises an auto-tune feature that improves accuracy and stability. It is applied in cases when the control result is unsatisfactory or for an initial set up of a new process. This function allows the controller to “learn” the process characteristics and automatically set the necessary control coefficients to minimize deviations of the measured values relative to the set point. In practical terms, this means that CO_2_ is injected in very short bursts and the controller waits for the system’s response (the evolution pattern of the measured CO_2_) before injecting more CO_2_. The CO_2_ is mixed with air (11) and injected into the header tank with an air pump (12) through air-stones (13) that create fine bubbles and allow a faster equilibration.

### Biological material

The thalli were collected by SCUBA diving in Armação de Pêra, Southern Portugal (37°011′.650″N/−8°19′.034″W) in a mäerl bed located at 20–25 m depth and 4.7 nautical miles from the coast. The algae were immediately transferred to a cool box and transported to the CCMAR’s (Centre for Marine Sciences) marine station in Ria Formosa coastal lagoon, southern Portugal. The thalli were gently cleaned to remove excess of epiphytes and kept in aquaria with filtered seawater under controlled conditions. The seawater was filtered through two in-line and two UV filters before entering the aquariums.

### Experimental conditions

Based on the International Panel on Climate Change (IPCC IS92) scenarios for atmospheric *p*CO_2_ increase (International Panel on Climate Change, 2000), three treatments were selected for this particular experiment: a control level running at current *p*CO_2_ (400 μatm) and two high CO_2_ levels (550 and 750 μatm). Six independent 25-L aquaria were used per treatment.

A hard plastic mesh platform was placed in the bottom of each aquarium, on top of which a ca. 5 cm-high layer of mäerl thalli was installed, mimicking the natural assemblage pattern observed in natural beds. The water entry tubing for each aquarium was placed beneath the mesh platform to guarantee circulation and prevent anoxic conditions. The thalli were regularly manually revolved in the aquaria, to emulate the natural movements of the algae in their seafloor beds. The aquaria were cleaned regularly to control the growth of turf algae.

In this particular experiment, the daily variability was minimal, in order to properly mimic the natural conditions of the natural mäerl beds where algae were collected. Temperature and irradiance levels were set to emulate as much as possible the prevailing conditions at the natural beds (where a long-term monitoring program is in place). Irradiance in the aquaria was set to mimic average irradiance values measured at 25 m depth in the mäerl bed where algae were collected, in Armação de Pêra, southern Portugal. The light source consisted of two green and white led strips of 24 W placed above each aquarium delivering a PAR of 8 μmol photons m^−2^ s^−1^. Photoperiod was adjusted seasonally using a timer to the desired L: D (light and dark, h) according to natural fluctuations. It varied from 10:14 in December to 15:9 in June. Temperature was allowed to follow the natural seasonal variation (14–20 °C).

### Seawater parameters

The *p*CO_2_ was continuously measured in the header tanks using an IRGA analyzer (WMA-4; PP Systems, MA, USA). Data was downloaded into a computer every 15 days. Salinity (CO310 conductivity meter; VWR, Radnor, PA, USA), pH (Orion 8103SC pH meter; Thermo scientific, Waltham, MA, USA), temperature (Roth digital thermometer; Hanna Instruments, Woonsocket, RI, USA) and dissolved oxygen (Symphony SB90M5, VWR, USA, accuracy ± 0.2 mg/L; ± 2%) were regularly monitored in the experimental aquaria.

TA was periodically measured at different points of the system; the 2,000 L source tank, the 200 L header tanks and the 25 L aquaria where the algae were kept. Water samples were poisoned with 20 μl of HgCl_2_ and sealed without bubbles in borosilicate Winkler bottles until analysis. TA was determined using the Gran titration method, as in Lewis & Wallace (1998). Sub-samples of 80 ml were titrated with HCl 0.5 M using an open cell automatic titrator (Metrohm 794; Metrohm, Herisau, Switzlerland). Alkalinity values were corrected using Certified Reference Materials (CRMs, Batches Nos. 121 and 126) supplied by A. Dickson (Scripps Institution of Oceanography, La Jolla, CA, USA). The carbonate chemistry of all water samples was determined from pH (measured with an Orion 8103SC pH electrode calibrated on the National Bureau of Standards (NBS) scale), TA, temperature and salinity using the CO_2_SYS software (Lewis & Wallace, 1998) with the constants of Mehrbach et al. (1973) (refitted by Dickson & Millero, 1987).

### Photosynthesis and respiration measurements

After 11 months of treatment with different *p*CO_2_ levels, algae were collected from the mesocosm for photosynthesis and respiration measurements. All measurements were conducted using water collected in the header tanks during three successive days, one day per CO_2_ level. Algae were maintained at controlled temperature at its respective CO_2_ level in a “walk in” chamber during the analyses.

A square section incubation chamber (15 ml volume) coupled to a Clark-type oxygen electrode (DW3/CB1; Hansatech, Norfolk, UK) was used for measurements of oxygen evolution (μmol O_2_·g FW^−1^·h^−1^). Photosynthesis-irradiance (P-I) response curves were built for all three *p*CO_2_ levels (400, 550 and 750 μatm) with six replicates per CO_2_ level. For each curve, eight light levels increasing from 6 to 860 μmol photons m^−2^ s^−1^ (PAR) were applied sequentially. The water temperature of the incubation chamber was set and maintained at 14 °C using a thermostatic bath with outer recirculation system (Julabo HC; Julabo Labortechnik, Seelbach, Germany). The maximum rate of photosynthesis (P_max_) at saturating irradiances was calculated for each curve using the model equation of Smith (1936).

For respiration rate measurements, six replicates were used per CO_2_ level (400, 550 and 750 μatm) and per each of eight temperatures (12, 14, 16, 18, 20, 22, 24 and 26 °C). Prior to the measurements, algae were acclimated at the tested temperature for two days in a “walk in” chamber where light and temperature are controlled. Measurements for each temperature were carried out the same day.

### Statistics

The software package SigmaPlot version 11.0 was used to perform the statistical analyses. Differences in respiration with *p*CO_2_ and temperature, and on photosynthesis with *p*CO_2_ and light were tested using two-way ANOVA tests. Normal distribution (Kolmogorov-Smirnov test) and equal variance (Levene’s test) were verified. When differences were significant (*P* < 0.05), ANOVA was followed by a post hoc test for multiple comparisons (Tukey’s HSD). Photosynthesis-irradiance curve fitting was performed using the SigmaPlot software package. Differences in P_max_ were tested using a one-way ANOVA test.

## Results

### CO_2_ control system assessment

**Figure 3:**
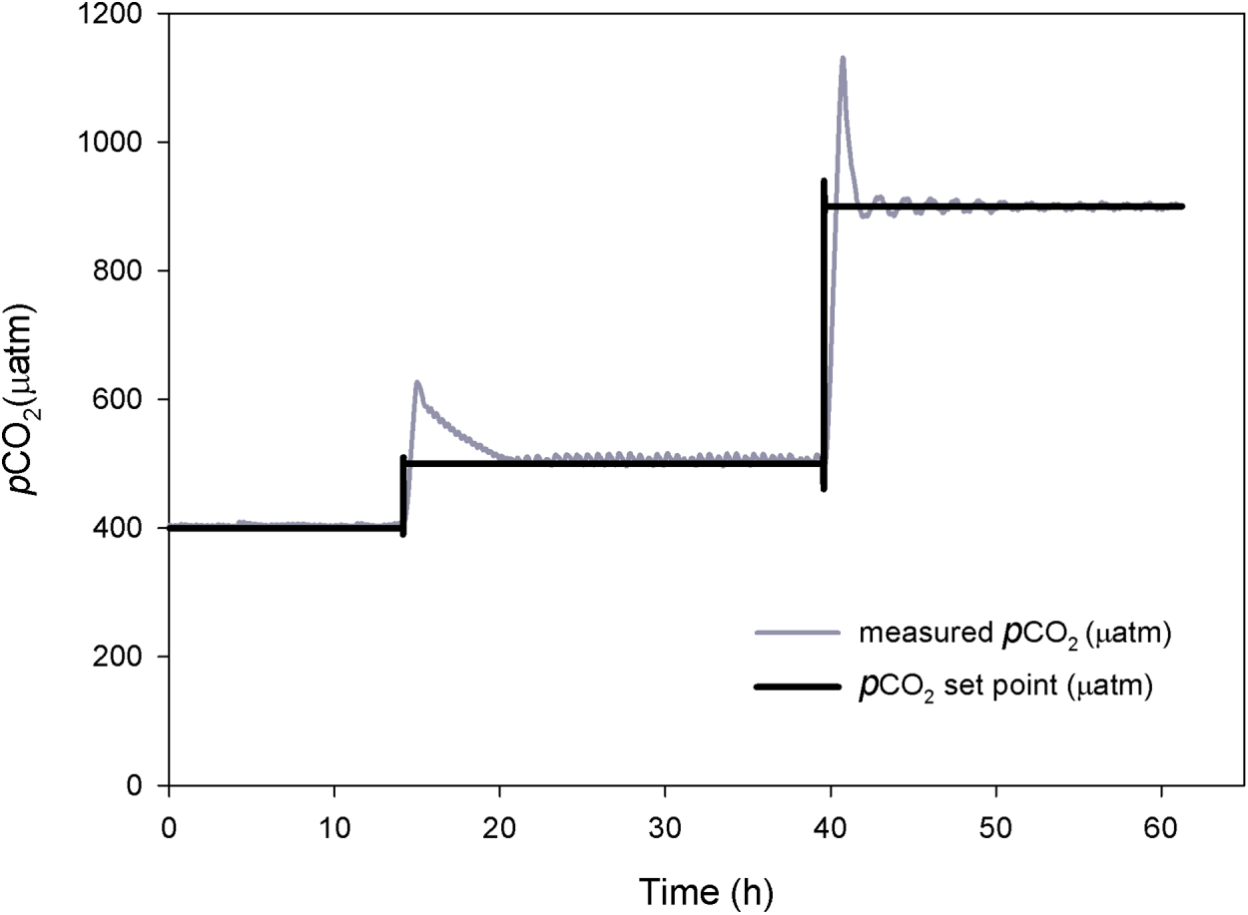
Control system testing. Response time (h) and stabilization of *p*CO_2_ (μatm). Preliminary tests at three different *p*CO_2_ levels.

The response time and performance of the system was evaluated at three different *p*CO_2_ levels. Each level took between two to five hours to reach steady-state (Fig. 3). At the higher *p*CO_2_ levels the system took longer to stabilize and experienced higher amplitude oscillations, requiring a frequent use of the auto-tune mode.

**Figure 4:**
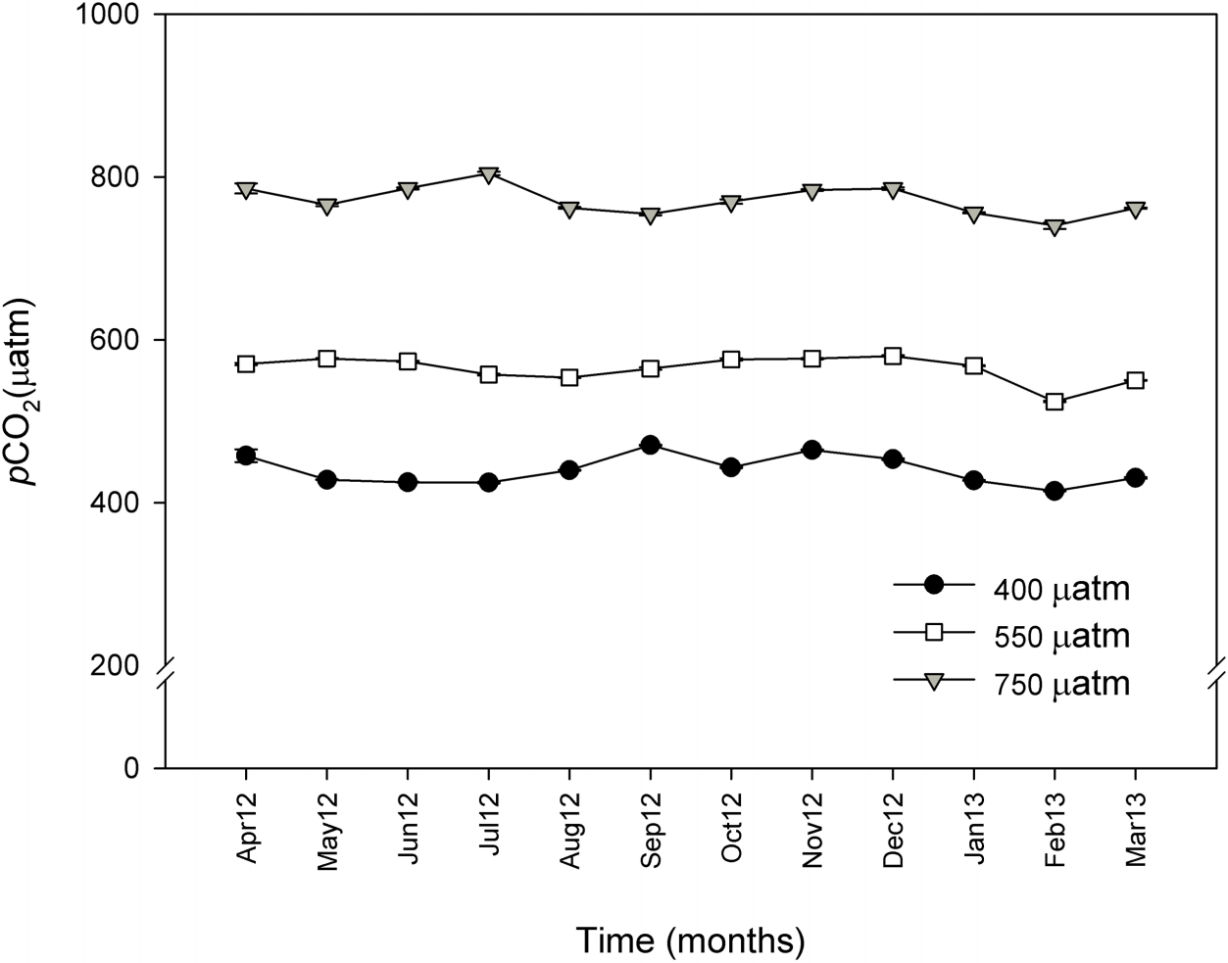
Monthly *p*CO_2_ values (μatm) during 11 months for control (∼400 μatm), 550 and 750 μatm *p*CO_2_ conditions. Monthly *p*CO_2_ values (μatm) from April 2012 (April 12) to March 2013 (March 13) for control (∼400 μatm), 550 and 750 μatm *p*CO_2_ conditions. The values were measured with an IRGA analyzer (WMA-4, PP-systems, USA) every 20 min and are expressed as mean ± SE.

The mean *p*CO_2_ values under control conditions ranged from 397 to 470 μatm of CO_2_. The mean values for the intermediate CO_2_ treatment went from 539 to 577 μatm of CO_2_, and the mean values for the high CO_2_ treatment went from 740 to 804 μatm of CO_2_. The control and the two enriched *p*CO_2_ levels in the system were consistent throughout the experimental period and reflected the daily variations observed under natural conditions (Fig. 4). TA ranged from 2,491.5 to 2,520.4 μmol. kgSW^−1^ and there were no significant differences between the sampled points of the experimental system and CO_2_ treatments. Table 1 shows the carbonate system after 11 months at the three different CO_2_ concentrations.

**Table 1:** Carbonate chemistry for each *p*CO_2_ level (400, 550 and 750 μatm) after 11 months of experimental treatment. Total alkalinity (TA), salinity, temperature and pH were measured while dissolved inorganic carbon (DIC), aragonite saturation state (Ωarag) and *p*CO_2_ were calculated using the CO_2_ SYS software. Values expressed as means ± SE (n = 11).

CO_2_ treatments	TA	S	T	pH	DIC	Ωarag	indirect *p*CO_2_
(μatm)	(μmol/kgSW)	(psu)	(°C)		(μmol/kgSW)		(μatm)
400	2,461.76 ± 4.08	34.87 ± 0.03	15.65 ± 0.19	8.18 ± 0.01	2,183.93 ± 7.48	3.09 ± 0.05	345.62 ± 7.31
550	2,449.17 ± 2.67	34.85 ± 0.02	15.93 ± 0.12	8.10 ± 0.00	2,215.27 ± 2.84	2.65 ± 0.03	430.60 ± 4.69
750	2,468.32 ± 2.84	34.63 ± 0.16	15.75 ± 0.14	7.90 ± 0.03	2,326.21 ± 11.02	1.81 ± 0.11	755.75 ± 56.41

The pH at the acidified treatments decreased gradually allowing for a proper acclimation of the algae. Temperatures in the aquaria during the experiment ranged from 13 to 19 °C, salinity ranged from 34 to 38 psu and dissolved oxygen was always close to 100% of saturation (∼6.7 mgO_2_/L).

### Long term effect of OA on coralline algae

**Figure 5:**
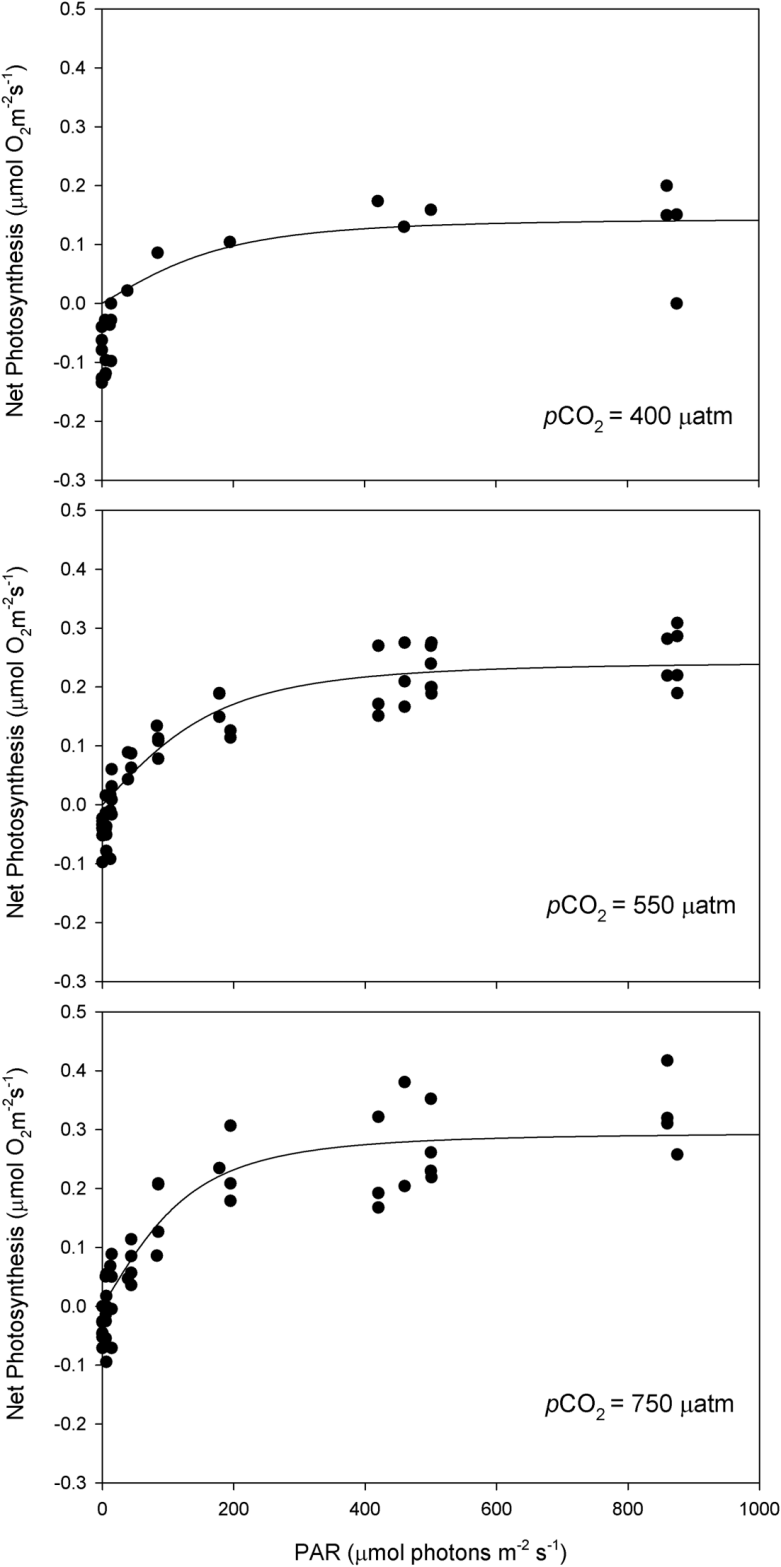
Light response curves of oxygen evolution with CO_2_. Light response curves of oxygen evolution (µmol O_2_·m^−2^·s^−1^) of *Phymatolithon lusitanicum*, under control (∼400 μatm), 550 and 750 μatm conditions, determined on individual thalli (n = 6) and fitted with the model equation of Smith (1936).

**Figure 6:**
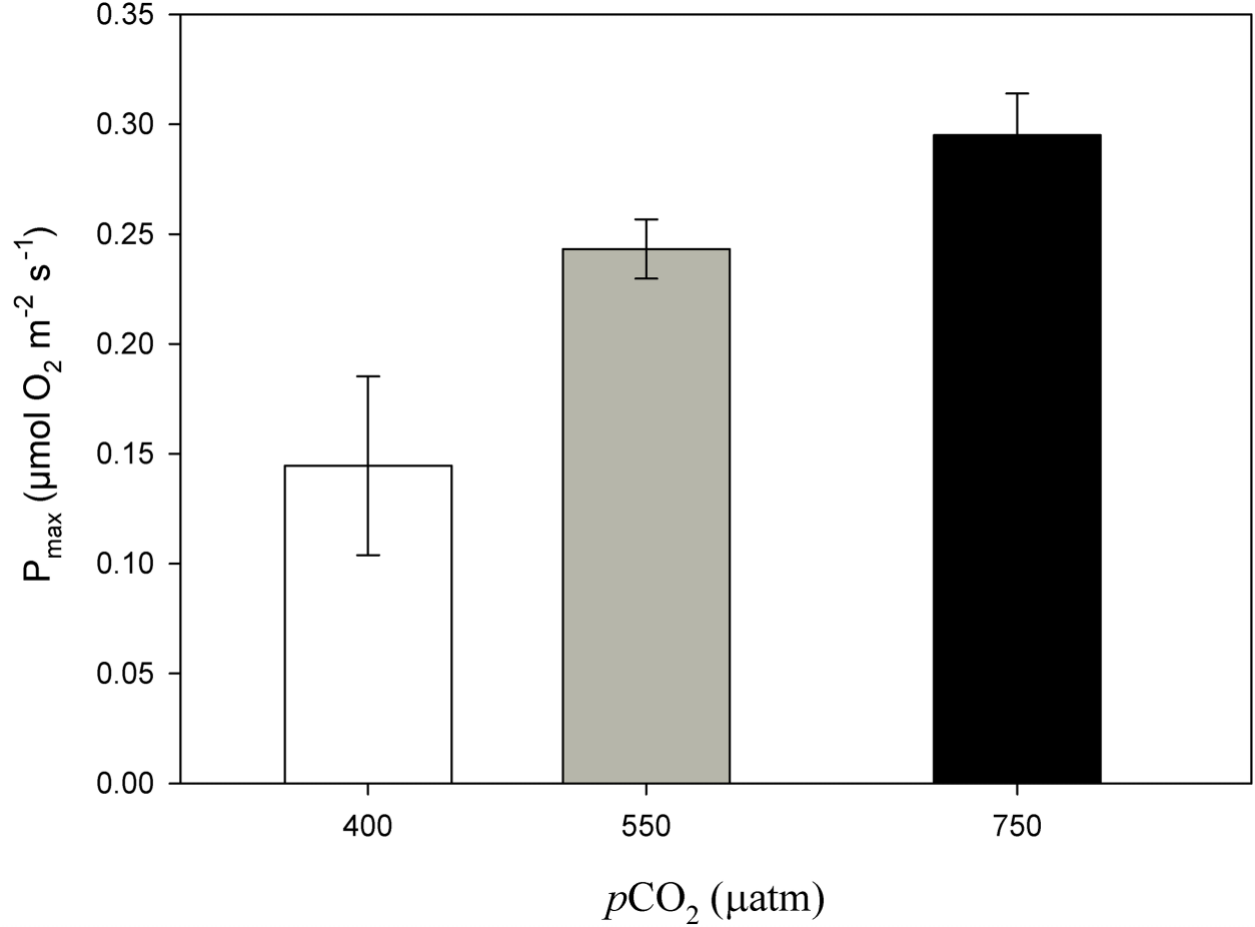
Maximum photosynthesis of *Phymatolithon lusitanicum* after 11 months at different CO_2_ levels. Maximum photosynthesis (P_max_) of *Phymatolithon lusitanicum* after 11 months at different CO_2_ levels (control ∼400, 550 and 750 μatm). Values expressed as mean ± SE.

**Figure 7:**
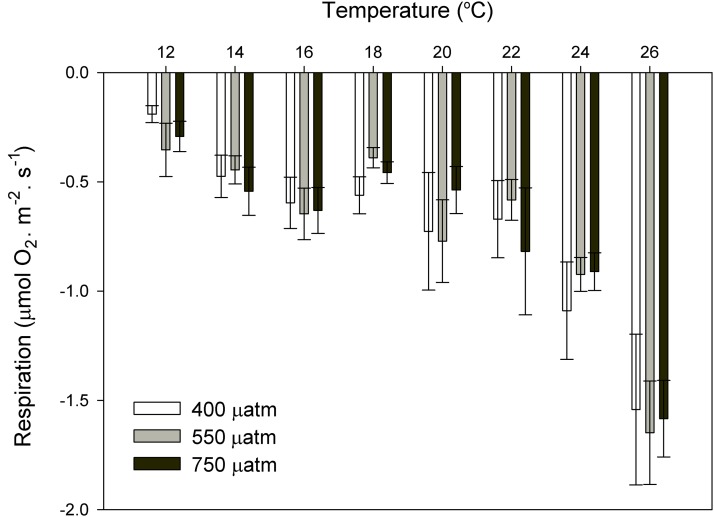
Respiration rates of coralline algae with CO_2_ and temperature after 11 months. Respiration rates (µmol O_2_·m^−2^·s^−1^) of coralline algae at different *p*CO_2_ (control ∼400, 550 and 750 μatm) measured in individual thalli using the Clark-type oxygen electrode at eight different temperatures from 12 to 26 °C with water exchange between replicates. Values expressed as mean ± SE (n = 6).

After 11 months of high *p*CO_2_ exposure, net photosynthesis was positively affected by elevated *p*CO_2_ and light (*P* ≤ 0.001). Results suggest that these algae have an irradiance threshold of 200 μmol photons·m^−2^·s^−1^ (PAR), above which photosynthetic rates saturate (Fig. 5). The maximum photosynthetic rates (P_max_) increased with *p*CO_2_ (*P* = 0.003) reaching the maximum values at 750 μatm of CO_2_ (0.29 μmol O_2_·m^−2^·s^−1^) with respect to 550 μatm (0.24 μmol O_2_·m^−2^·s^−1^) and control conditions (400 μatm) (0.14 μmol O_2_·m^−2^·s^−1^) (Fig. 6). Respiration increased with temperature (*P* ≤ 0.001) but it was unaffected by CO_2_ (*P* = 0.965). The highest respiration rates (μmol O_2_·m^−2^·s^−1^) were observed at 26 °C and the lowest at 12 °C (Fig. 7).

## Discussion and Conclusions

This mesocosm is equipped with a simple, accurate and reliable control system that operates almost unattended apart from the regular maintenance and data download routines. Instead of using pH, the system uses CO_2_ as a control variable, measured with an accurate CO_2_ analyzer coupled to a PID controller. This combination circumvents the problems and uncertainties associated with pH control and allows the maintenance of long-term stability in the set *p*CO_2_ values. This allows a proper acclimation of the organisms to different CO_2_ levels and a more realistic response of their metabolic rates. It is also possible to test other relevant variables simultaneously with CO_2_, such as temperature, irradiance, nutrients etc.

The combination of the gas-flushing equilibrator and the air pump results in an improved gas mixing, taking the system only 2–5 h to reach a steady state. The gas-flushing equilibrator is a simple structure, easy to build and with a short response time, that can be used in highly turbid waters (Frankignoulle, Borges & Biondo, 2001) and CO_2_ oversaturated water (Frankignoulle & Borges, 2001). Even if an air pump to bubble the experimental water is not commonly used in OA studies, it has been suggested by Gattuso & Lavigne (2009) as a simple alternative to inject the CO_2_-air mix. In this experiment, this combination has proven to be efficient.

In addition, this gas bubbling system is more economical than previous designs that use pre-mixed gasses, which are expensive in long-term experiments and only suitable for small volumes (Gattuso et al., 2010). Once the system is stabilized, the consumption of food-grade 100% CO_2_ is largely minimized by the reduction of gas waste obtained with the PID control, making the system not only suitable but also affordable for long-term experiments (months to years).

This control system can also be adapted to other types of organisms, namely phytoplankton, since there is no direct bubbling into the experimental tanks. Seawater aeration by bubbling might lead to difficulties in phytoplankton cultures because it may enhance the coagulation of organic matter (Gattuso & Lavigne, 2009) or damage the calcareous structures.

It is now widely accepted that OA experiments should incorporate the natural patterns of daily fluctuations in pH. This is particularly relevant when studying species that live in environments with considerable variability, such as shallow marine habitats or coastal upwelling systems (see Eriander, Wrange & Havenhand, 2016; Reum et al., 2015). With this control system, the habitat-specific *p*CO_2_ variability can be easily incorporated in the control routine, in a similar way than with pH stats (see Eriander, Wrange & Havenhand, 2016). The PID controller can be programmed to adjust the CO_2_ injection along the day, as a function of both the target value and its desired degree of daily fluctuation.

Following the recent recommendations of Cornwall & Hurd (2016), we have in the meantime adapted the experimental design depicted in Fig. 2. Instead of using the header tanks to mix the CO_2_ directly with seawater, CO_2_ is injected into a large air tank (4,000 L), where it is mixed with air to obtain the target *p*CO_2_ value. This target value is set and controlled by a PID controller coupled to a gas analyser (IRGA). The pre-prepared mixture is then pumped by an air compressor and injected in the header tanks, one per experimental tank. The CO_2_-enriched air is equilibrated with seawater in the header tanks using air stones. In this way, all the experimental tanks are true replicates. This experimental design has already been used effectively in an experiment with the seagrass species *Cymodocea nodosa* (M. Ruocco et al., 2016, unpublished data).

The photosynthetic and respiration results after 11 months of high CO_2_ exposure showed that this system is adequate to run long-term experiments with coralline algae and can be easily adapted to be used with other organisms. The increased rates of maximum photosynthesis (P_max_) under elevated *p*CO_2_ indicate higher carbon uptake via photosynthesis with the increase of CO_2_ concentration. After 11 months of high *p*CO_2_ exposure, *P. lusitanicum* revealed physiological capacity to acclimate to high *p*CO_2_. This agrees with the results from Noisette et al. (2013a) and Noisette et al. (2013b), in which the photosynthetic rates of the mäerl species *Lithothamnium corallioides* increased with CO_2_. However, Martin et al. (2013) and Martin & Gattuso (2009) found decreasing or unaffected photosynthetic rates of the crustose coralline algae *Lithophyllum cabiochae* after one year of exposure to high CO_2_. Time of acclimation and exposure is an important factor which could partially explain the differences found among OA experimental studies where coralline algae increase, decrease or have a parabolic photosynthetic response to OA (reviewed in Martin et al., 2013). The results from this study suggest that more long-term studies with coralline algae are needed. It appears important to gradually acclimate the algae to acidified conditions and compare the results after at least two different time periods. However, it is also important to consider that the responses in long-term experiments might not be necessarily indicative of those that will occur in the future. Martin & Gattuso (2009) found that after one year in the laboratory the calcification rates of the crustose coralline alga *Lithophyllum cabiochae* decreased by several orders of magnitude. The authors attributed this decrease to the stress caused by changing environmental conditions from natural to artificial ones. The interaction with natural seasonal fluctuations of environmental parameters such as irradiance and temperature are determinant factors in OA experiments. To properly mimic the natural conditions, we suggest that experimental studies should be accompanied by monitoring programs in the field.

Respiration rates responded to temperature but not to CO_2_, suggesting that in a future ocean, the respiration rates of these coralline algae from southern Portugal will increase because of ocean warming, independently of OA. This agrees with previous OA studies where respiration in coralline algae was unaffected by CO_2_ (Noisette et al., 2013a) but positively affected by temperature (Noisette et al., 2013b; Martin et al., 2013).

Photosynthesis and respiration results suggest that temperature and irradiance have an important role on the metabolism of coralline algae (see Vásquez-Elizondo & Enríquez, 2016; Martin et al., 2013) and should be carefully controlled and considered in OA experiments. Moreover, the effect of temperature on respiration is key to assess the effects of climate change in these organisms, as it will be reflected on the global carbon budgets of mäerl beds. The direct control of *p*CO_2_ is probably the best way to standardize comparisons among high-CO_2_ experiments (see Reum et al., 2015). Since similar control systems are already being used in terrestrial and freshwater ecosystems, the use of a unique control system not only facilitate the inter-comparisons but would also allow more reliable estimates of the impacts of high *p*CO_2_ in the global carbon fluxes.

Many questions remain unanswered in OA research and the inconsistencies and differences in results due to technical problems must be minimized namely through the improvement of experimental setups and standard protocols. We demonstrated that a direct control of CO_2_ can be used in long term OA experiments and propose this simple and affordable control system that is also flexible enough to be adapted and customized according to specific requirements.

## Supplemental Information

10.7717/peerj.2503/supp-1 Supplemental Information 1 Raw data from Figs. 3–7 and Table 1.

## Additional Information and Declarations

### Data Deposition

The following information was supplied regarding data availability: The raw data has been supplied as Supplemental Dataset Files.
